# Temperature and Strain Characterization of Tapered Fiber Bragg Gratings

**DOI:** 10.3390/s25247520

**Published:** 2025-12-11

**Authors:** Camila Carvalho de Moura, Valmir de Oliveira, Hypolito José Kalinowski, Claudecir Ricardo Biazoli

**Affiliations:** 1 CPGEI—Programa de Pós-Graduação em Engenharia Elétrica e Informática Industrial, Universidade Tecnológica Federal do Paraná, Curitiba 80230-901, Brazil; valmir@utfpr.edu.br; 2 Departamento acadêmico de Engenharia de Telecomunicações, Universidade Federal Fluminense, Niterói 24220-900, Brazil; hjkalinowski@id.uff.br; 3 Instituto de Física Gleb Wataghin, Universidade Estadual de Campinas, Campinas 13083-859, Brazil; claudecirbiazoli@gmail.com

**Keywords:** fiber Bragg grating, strain sensitivity, tapered fiber Bragg grating

## Abstract

This work presents a systematic experimental investigation of tapered fiber Bragg gratings (tFBGs) fabricated from standard SMF-28 fiber with waist diameters ranging from 30 to 115 µm. The effects of taper geometry on strain and temperature sensitivities were evaluated using UV inscription through two phase masks to ensure reproducibility. The maximum strain sensitivity achieved was 25.38 ± 0.06 pm/N for the 30 µm waist, corresponding to 20.84 ± 0.05 pm/µε—an enhancement of more than 1600% compared to a standard untapered FBG. In contrast, the thermal sensitivity remained nearly constant at ~12.5 pm/°C for all diameters, confirming that the temperature response is governed by the intrinsic thermo-optic and thermal-expansion properties of silica and is not significantly affected by taper geometry. The measured strain sensitivity exhibited a clear inverse-square dependence on the waist diameter, in excellent agreement with a simple axial-stress model. Consistent Bragg responses obtained using different phase-mask pitches further validated the repeatability of both the tapering and inscription processes. These results demonstrate that tapering standard telecom fiber provides a low-cost, scalable, and robust method to significantly enhance FBG strain sensitivity while preserving thermal stability, enabling compact and high-performance sensors for structural and industrial monitoring applications.

## 1. Introduction

Fiber Bragg gratings (FBGs) are a well-established sensing technology widely used in structural health monitoring, aerospace, civil engineering, and energy systems due to their compactness, electromagnetic immunity, and multiplexing capability [1,2,3]. In standard single-mode silica fibers such as SMF-28^®^, the intrinsic strain sensitivity of FBGs is limited by the material’s mechanical and photoelastic properties, motivating the development of techniques to enhance sensitivity [1,2,4]. Several approaches have been reported to improve FBG responsiveness, including the use of specialty fibers [5], femtosecond-laser inscription [6], chemical etching [7,8,9], and geometric modification through tapering or micro/nanofiber drawing [10,11,12,13,14,15]. Although approaches based on exotic fibers [5,6] or ultrafast inscription can operate in harsh environments [6,14], they typically involve higher cost and more complex fabrication procedures, while chemically etched gratings [7,8,9] often require careful processing and may introduce structural non-uniformities. Tapering standard SMF-28 fibers has emerged as a simple and scalable method to enhance FBG strain sensitivity [12,15,16]. By reducing the cladding diameter in a localized waist region, the cross-sectional area decreases, resulting in a larger wavelength shift per applied force [7,13,14]. In addition, tapering modifies the modal confinement of the guided mode, influencing the Bragg wavelength [5,10,11,13]. Importantly, because the grating is inscribed in the silica waist, the thermal sensitivity remains governed by the thermo-optic and thermal-expansion coefficients of silica—leading to an almost geometry-independent temperature response [1,2,7,13]. Despite several demonstrations of taper fabrication and applications in sensing [11,12,13,15], a systematic experimental investigation quantifying how taper diameter influences both strain and temperature sensitivities remains limited [7,13,14]. In particular, the scaling of strain sensitivity with diameter reduction and the reproducibility of UV-inscribed gratings in tapered regions require further clarification [1,15,17]. In this work, we experimentally characterize tapered FBGs (tFBGs) fabricated from SMF-28 fibers with waist diameters ranging from 30 µm to 115 µm. Using two UV phase masks, we evaluate reproducibility and analyze the dependence of strain and thermal sensitivities on taper geometry. We show that strain sensitivity increases predictably with the inverse square of the diameter, while thermal sensitivity remains nearly constant (~12.5 pm/°C) across all tapers. The results validate tapering as a low-cost and practical route to achieve high-sensitivity FBG-based strain sensors without compromising thermal performance. Finally, comparisons with representative recent works, including half-etched [8,9] and sapphire FBGs [6], highlight the advantages and application potentials of the proposed approach.

## 2. Materials and Methods

The FBG shows a reflected spectrum with a characteristic Bragg wavelength (λ_B_), as given by [1,2,4,17]:

λ_B_ = 2Λn_eff_ (1)

where *n_eff_* is the effective refractive index of propagated mode and Λ is the grating pitch. When a phase mask is used under direct UV illumination in FBG recording, with the optical fiber positioned under the phase mask, Λ is determined by phase mask pitch (Λ_PM_) [1,2,4,17]:

Λ = Λ_PM_/2 (2)

The FBG is intrinsically a temperature and strain sensor. Fiber Bragg grating sensors offer a comprehensive sensing platform due to their sensitivity, selectivity, and compactness [1,2,7,13]. FBG-based sensors have been successfully applied for tunnel excavation monitoring [18], demonstrating their versatility in geotechnical environments. FBG sensors have also been employed in rock bolt support monitoring systems [18].

Alternative optical sensors as long-period fiber gratings (LPGs) have also been explored for fluid level and velocity monitoring, with promising results in biological and chemical sensing [19,20] and Plasmonic Mach-Zehnder interferometers have been used for gas sensing in mid-infrared regimes [21].

The temperature/strain variations (with crossed sensitivity) change the refractive index of the medium, which affects the effective index of the propagation, and the Bragg grating spatial pitch. Therefore, a shift in the Bragg wavelength occurs (Δλ_B_), which can be related to temperature/strain for measurements and sensor applications. The external parameters changes induce axial grating pitch (Δ*l*) or temperature (ΔT) variations, resulting in (Δ*λ_B_*), as shown in [1,2,4,17]:

Δλ_B_ = 2[(Λ∂n_eff_/∂*l*)+(n_eff_∂Λ/∂*l*)]Δ*l* + 2[(Λ∂n_eff_/∂T)+(n_eff_∂Λ/∂T)]ΔT (3)

The term (Λ∂n_eff_/∂T) is due to the thermo-optic effect, whereas the term (n_eff_∂Λ/∂T) caused by the thermal expansion.

The final term (Λ∂n_eff_/∂*l*) is due to the elastic-optic effect. Simultaneous strain and temperature discrimination can be achieved using a combination of FBGs and Brillouin scattering [22], Bragg gratings in microstructured fibers [5] exhibit distinct behavior when compared to standard fibers, particularly for temperature-strain separation, Modal interferometry in Bragg fibers [23] has been used for strain and temperature separation with high sensitivity. Single tilted fiber Bragg gratings [24] have been demonstrated for effective discrimination between temperature and strain. Recent research has highlighted the potential of speckle-based [25], plasmonic [21,26], and micro-interferometric techniques [23] for refractive index and pressure sensing. However, FBG-based systems remain attractive due to their multiplexing capability, compactness, and maturity.

### 2.1. Tapered Fibers Production

Tapers were fabricated through the heating of the optical fiber using an electric resistance system [12,15,16,27]. This heating method enables greater control of process temperature [28], ensuring better reproducibility compared to flame-based methods [27], which may affect fiber composition due to the presence of combustible gases. It is also more cost-effective than laser-based heating systems. At 1200 °C the fiber viscosity is low enough to allow for stretching.

**Figure 1:**
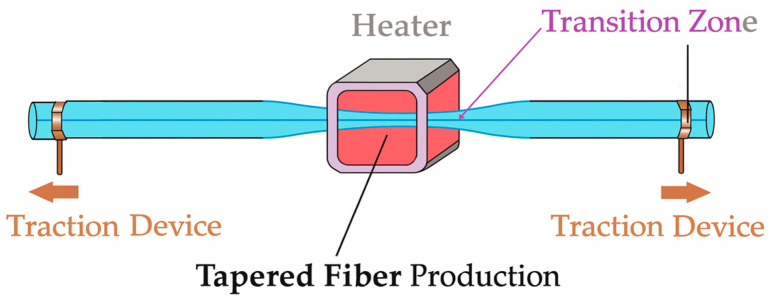
Fabrication procedure of the tapered fibers using the heat-and-pull technique. A controlled flame is applied to soften the silica while the fiber is symmetrically stretched by motorized stages, producing a uniform waist with smooth transition regions. This process enables precise control of the final diameter for subsequent FBG inscription.

Tapered optical fibers were fabricated using a standard heat-and-pull technique [12,15,16,29] with precise control of the final waist diameter. A section of SMF-28 fiber was stripped over ~40 mm, cleaned with isopropyl alcohol, and mounted on a computer-controlled tapering rig composed of two precision linear translation stages (Thorlabs Z825B, Newton, NJ, USA). The fiber was gently tensioned to remain straight without introducing pre-strain, and the center of the stripped region was aligned with a ceramic micro-heater (1 mm^2^ Ni-Cr resistive element) featuring a 1.5 mm hot zone. The heater temperature was regulated at approximately 1200 °C using a closed-loop PID controller, providing stable and uniform heating [29]. Once thermal stability was reached, the stages pulled symmetrically at 0.03 mm/s [28], softening and elongating the silica to form adiabatic tapers [16], as seen in Figure 1. The final waist diameter (30–115 µm) was determined by the programmed pulling distance, following the standard exponential tapering model [16] *D_f_* = *D*_0_*e*^−^*^L^*^/2^*^z^*, where *D*_0_ = 125 μm. Each taper consisted of a uniform waist (~10 mm) and two symmetric transition regions (~15 mm each). After pulling, the fiber was allowed to cool naturally for approximately 60 s under constant tension to avoid residual stress and ensure smooth, cylindrical geometry. The resulting structures were inspected under a digital optical microscope, and the waist diameter was measured at three axial positions; only tapers exhibiting <3% diameter variation were accepted [30]. Repeated fabrications at each target diameter confirmed a mean deviation below 3%, demonstrating the reproducibility and stability of the tapering process. Accepted tapers were subsequently transferred to the UV-inscription setup without contacting the waist region, where the FBG was written using a 193 nm ArF excimer (Coherent Xantos Xs excimer laser model XS-L 500-193 nm, Santa Clara, CA, USA) laser through a phase mask covering the entire waist [2,4,17].

Considering the laminar flow of the glass during the taper manufacture, it is expected to see a proportional reduction between cladding and core diameters [16]. The obtained tapers have cladding and core diameters from 125 µm/8.2 µm to 30 µm/1.97 µm, respectively. The final taper diameters were determined by optical microscopy after production. The taper transition profile is irrelevant since the FBG is written in the waist zone [29,30]. The variation in the taper waist diameter directly affects the modal properties of the fiber and consequently modifies the reflected spectrum of the Bragg grating [5,10,11]. As the waist diameter decreases, the confinement of the fundamental mode becomes weaker, which reduces the effective refractive index (n_eff_) and leads to a blue shift in the Bragg wavelength [10,11,16]. At the same time, the reduced diameter decreases the grating coupling coefficient due to reduced modal overlap with the refractive-index modulation, resulting in lower reflection intensity for smaller diameters [5,10,11]. In contrast, the spectral bandwidth remains essentially unchanged because the FBG is inscribed entirely within the uniform waist region, where the diameter is constant and no chirp is introduced [2,17].

**Figure 2:**
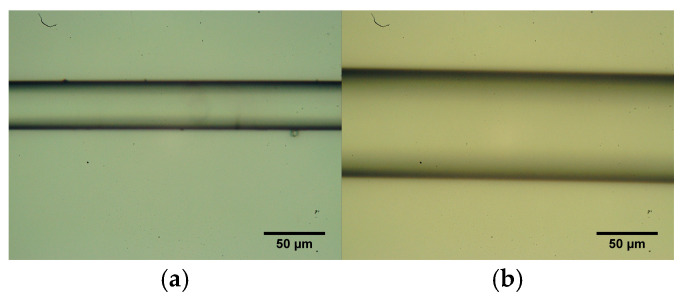
Optical micrographs of the tapered waist region for (**a**) 40 µm and (**b**) 80 µm diameters. Images were acquired with a digital optical microscope immediately after the tapering stage. The dashed box indicates the uniform waist section where the FBG was inscribed. Scale bars correspond to 50 µm. The uniformity and smoothness of the waist region ensure adiabatic mode propagation and consistent grating inscription.

Images of the tapered region (40 µm and 80 µm diameters) by optical microscopy, from previous studies are available and referenced in earlier work [15], as shown in Figure 2.

The produced tapers have lengths ranging from a few centimeters to several tens of centimeters, which is inversely proportional to the taper diameter due to the conservation of volume during fiber drawing [16].

Standard (Corning SMF28/G652, Wilmington, NC, USA) optical fiber has a strain limit of approximately 1% (10,000 µε) [31]. However, the excimer laser writing process alters the fiber structure, which may reduce this limit [4,17,32]. For tapered fibers with inscribed Bragg gratings, this limit is likely lower [14,30], although specific studies on this aspect were not performed here. Related mechanical behavior under strain is addressed in [8,14].

### 2.2. Writing Process

After tapering, the FBG inscription was performed using an ArF excimer laser (Coherent Xantos Xs excimer laser model XS-L 500-193 nm, Santa Clara, CA, USA) and the phase-mask technique [4,17,32]. Two phase masks were employed to verify the repeatability of the inscription process and to produce slightly different Bragg wavelengths for comparative analysis. The laser fluence was adjusted to maintain consistent refractive-index modulation across all samples [2,17]. The grating length was approximately 3 mm, corresponding to the uniform waist section of each taper.

**Figure 3:**
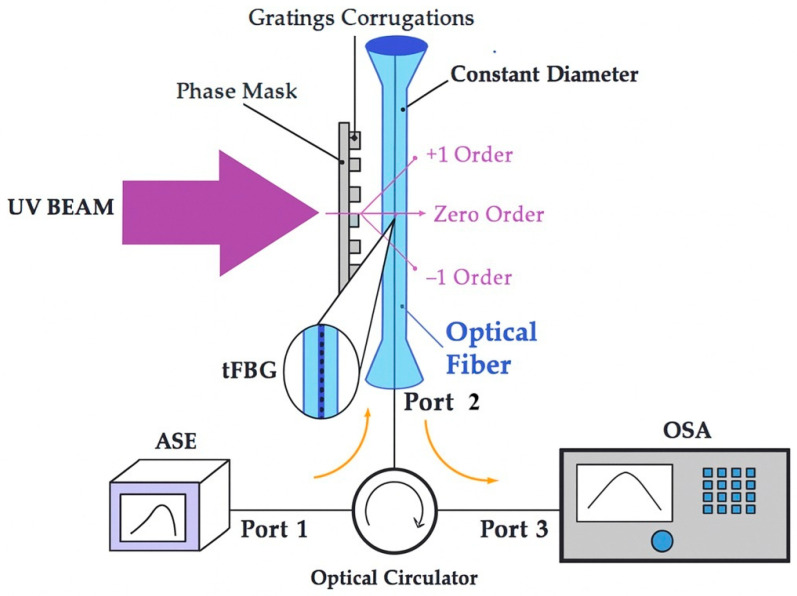
Schematic diagram of the tFBG inscription setup and interrogation circuit. A 193 nm excimer laser illuminates the fiber through a phase mask to generate the periodic index modulation, while an optical spectrum analyzer (OSA) continuously monitors the Bragg resonance during inscription. The configuration ensures precise alignment and stable inscription across the tapered waist.

The method used to write the tapered FBGs (tFBGs) was illumination under a phase mask, using two different uniform phase masks with pitches Λ_PM1_ = 1058.9 nm and Λ_PM2_ = 1064.9 nm, both optimized for operation at 193 nm [17,32]. There are various methods to write FBGs in standard optical fibers without enhancing photosensitivity, such as phase mask techniques [17,32] and excimer laser exposure [4,32]. The UV laser beam source was Coherent Xantos Xs excimer laser model XS-L 500-193 nm, Santa Clara, CA, USA. A broadband light source ALS-10-M Amonics^®^ ASE (Hong Kong, China) (amplified spontaneous emission) module, output power 10 dBm and operation in the C-band, illuminated the fiber through an optical circulator (Thorlabs^®^ 6015-3 FC/PC, Newton, NJ, USA) to measure spectra with an optical spectrum analyzer (OSA)—(Yokogawa^®^ AQ6375, Tokyo, Japan) with 0.02 nm resolution, as shown in the setup on Figure 3. All measurements were performed at room temperature (22 ± 1 °C) unless otherwise specified. Prior to characterization, the fibers were cleaved and connected to minimize insertion loss.

The utilization of two different phase masks (resulting in two distinct Bragg wavelengths) was carried out to validate the generality of our core findings. By demonstrating that the predicted D^−2^ strain sensitivity scaling holds true regardless of the absolute Bragg wavelength or grating period, we confirm that the mechanism of sensitivity enhancement is solely governed by the reduced geometric profile of the fiber [2,17].

The gratings were written in the smaller and constant diameter regions in the tapered fiber (waist zone), where the mode confinement is well-defined [14,15]. All tFBGs have a length of about 3 mm, because the laser spot size determines the grating length, a well-established feature of excimer-based FBG inscription [2,17]. Similar observations were reported in previous studies involving tFBG fabrication using excimer sources [14,15], recorded using a repetition rate 250 Hz laser, energy 2.5 mJ/pulse and 2 min exposure time. The tapers diameters and numbers from pitch Λ_PM_ = 1064.9 nm are shown in Table 1.

The number of samples for each taper diameter varied depending on fabrication availability. The uniformity in grating inscription was ensured by selecting stable regions of constant diameter [15,16].

**Figure 4:**
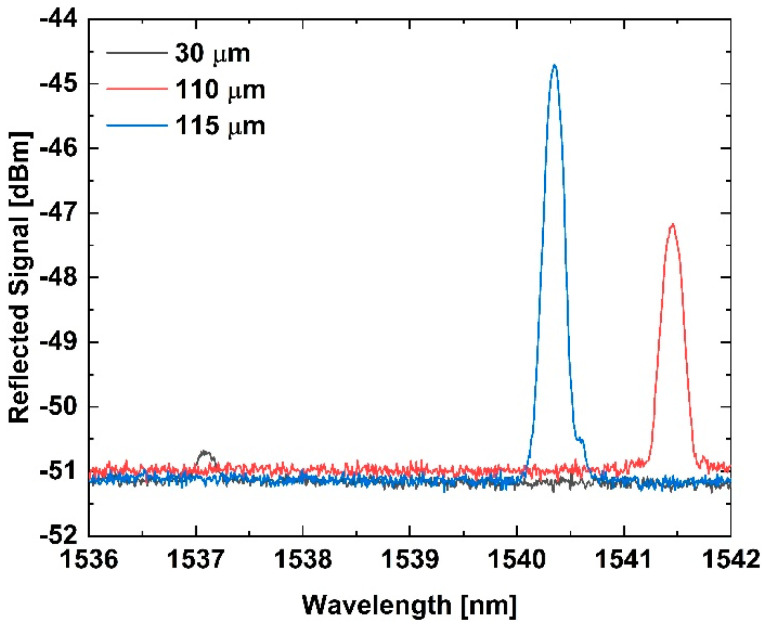
Bragg wavelength for tFBGs with different waist diameters. The spectral shift observed between samples results from the variation in the effective refractive index (n_eff_) with the cladding diameter. As the fiber becomes thinner, n_eff_ decreases, leading to a shorter Bragg wavelength according to (1).

The tFBGs were written only in the narrow waist, which avoids the chirping effect due to the uniform diameter [2,13,17]. Figure 4 shows two tFBGs reflection spectra to 110 µm and 115 µm waist diameter. Tapered regions were symmetrical and reproducible [12,15,29,30]. Chirped Bragg gratings in tapered fibers allow for better discrimination between thermal and mechanical effects [13].

We noted that the mass-loading method provides a highly linear and reproducible application of load for comparative study across the wide range of fiber diameters. The conversion from load to strain assumes a uniform stress distribution, which is valid because the FBG inscription and subsequent characterization are confined solely to the uniform, constant-diameter waist region of the tapered fiber [14,30,31].

### 2.3. Thermal Characterization

**Figure 5:**
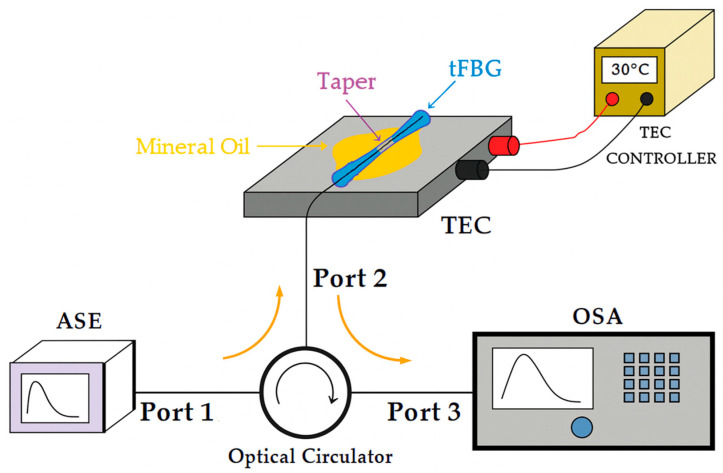
Experimental setup used for thermal characterization of the tFBGs. The tapered region is immersed in a sealed quartz cuvette filled with mineral oil to minimize convection and humidity effects. A PID-controlled heating module ensures temperature stability, while the Bragg wavelength is monitored in real time using an OSA. A 10 min stabilization period was used before each measurement point.

Thermal sensitivity for tFBGs written with pitch Λ_PM2_ = 1064.9 nm was investigated by placing the samples in a programmable TEC (thermoelectrical cooler) with ±0.1 °C temperature stability, as schematically showed in Figure 5. The temperature varied from 10 °C to 70 °C in 10 °C increments. At each step, the Bragg wavelength was monitored after a 10 min stabilization period to ensure thermal equilibrium. For efficient thermal coupling between the TEC and the taper, mineral oil is used at the interface.

Direct comparison of the Bragg spectrum before and after applying mineral oil—recorded using the OSA’s trace-comparison function—showed no change in wavelength, spectral shape or peak contrast. This demonstrates that, even at a 30 µm waist, the guided mode remains strongly confined to the silica structure, with negligible influence from the external medium [5,10,11].

The thermal sensitivity S_temp_ (pm/°C) was calculated from the linear fit of Δλ_B_ versus temperature, expressed as follows [1,2,7]:

S_temp_ = dλ_B_/dT = λ_B_(α + ξ) (4)

where α is the thermal expansion coefficient and ξ is the thermo-optic coefficient of silica [1,2,7]. The measured sensitivity remained approximately constant (~12.5 pm/°C) for all diameters, indicating that the thermo-optic contribution dominates and that the taper geometry does not significantly influence the temperature response [1,2,7,8].

### 2.4. Strain Characterization

**Figure 6:**
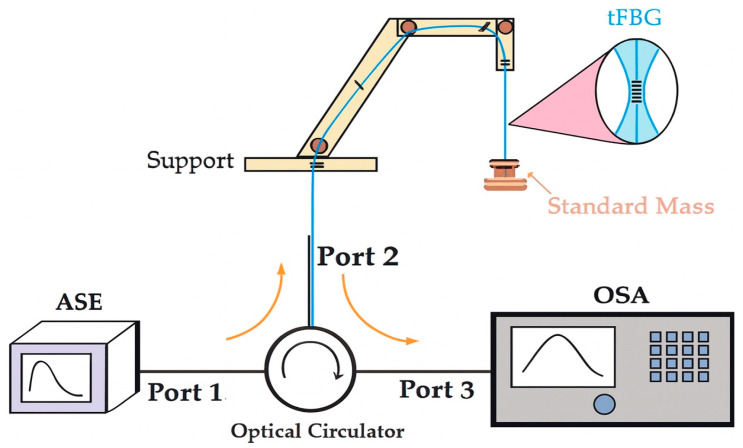
Setup used for strain characterization through suspended mass loading. The mass is attached to the free end of the fiber, producing pure axial tension on the tFBG waist. The unprocessed fiber sections are fixed to precision clamps to avoid bending or shear. Bragg wavelength shifts are recorded as a function of the applied axial load.

Strain sensitivity measurements were conducted using a simple mass-loading setup, as illustrated in Figure 6. One end of the fiber was fixed to a support, while calibrated weights were attached to the other end to generate controlled axial forces. Each measurement had 10 min stabilization time, and the used masses were 1 g, 2 g, 5 g, 10 g and 20 g. The applied load was converted to force (F = mg) and then to strain in the waist region assuming uniform stress distribution, as the grating was located entirely within the constant-diameter section [30,31]. The gravitational acceleration at the test site was 9.78 m/s^2^.

The Bragg wavelength shift (Δλ_B_) was recorded as a function of the applied load using the OSA. Strain sensitivity was determined from the slope of the linear regression Δλ_B_ = S_load_ × F, where S_load_ represents the wavelength shift per unit force (pm/N). The corresponding strain sensitivity (pm/µε) was obtained using the Young’s modulus of silica (E = 72 GPa) [31] and the cross-sectional area A = π (D/2)^2^. The uncertainty in each measurement was estimated from repeated load-unload cycles and instrumental resolution, resulting in error bars of ± σ for all plotted data.

Although the mass-loading method provides limited precision compared to micro-tensile systems, it offers good repeatability and simplicity for comparative studies across multiple fiber diameters [14,15,30]. The accuracy was further confirmed by comparing the results obtained using the two-phase masks, which yielded nearly identical sensitivities [2,14,15,17].

The applied weight can also be expressed in terms of strain by using the relation [30,31]:

F = εEA(5)

where *E* is the Young’s modulus of silica (≈72 GPa) and *A* is the fiber cross-section area [31]. Thus, 1 µε corresponds to approximately 0.2 mN for a 60 µm fiber, allowing for direct conversion between mechanical load and strain measurement in the tapered region [30,31,33].

The observed enhancement in strain sensitivity with decreasing fiber diameter is a fundamental consequence of basic mechanical principles, as the effective sensing area is reduced. The relationship between applied force (F), axial stress (σ), and strain (ε) is defined by σ = F/A and ε = σ/E, where A = π (D/2)^2^ is the cross-sectional area and E is the Young’s modulus [30,31]. Under a constant applied force (F), the strain (ε) is inversely proportional to the square of the fiber diameter (D) [30,31].

ε ∝ (F/D^2^) (6)

Since the Bragg wavelength shift (Δλ_B_) is directly proportional to strain (Δλ_B_ = S_ε_ ε), a reduction in diameter D leads to a corresponding squared-inverse increase in strain for a given force [1,2]. Therefore, the load sensitivity (S_load_ = Δλ_B_/ΔF) scales as D^−2^, and this is the dominant factor in our experimental observations [30]. This analysis allows us to directly link the measured load sensitivity in pm/N to the intrinsic strain sensitivity in pm/µε using S_ε_ = S_load_(AE)^−1^, with E = 72 GPa for fused silica [31].

Furthermore, the wavelength shift observed between different diameter fibers (e.g., Figure 4) is primarily due to the change in modal confinement. As the diameter decreases, the mode field expands further into the cladding, reducing the effective refractive index (n_eff_) of the guided mode and causing a blue shift in the Bragg wavelength (λ_B_ = 2Λn_eff_) [10,11,27,28]. This expansion enhances the interaction with the surrounding medium, which is utilized for sensing, while the adiabatic taper profile ensures minimal coupling loss [16,27].

## 3. Results

### 3.1. Thermal Sensitivity

**Figure 7:**
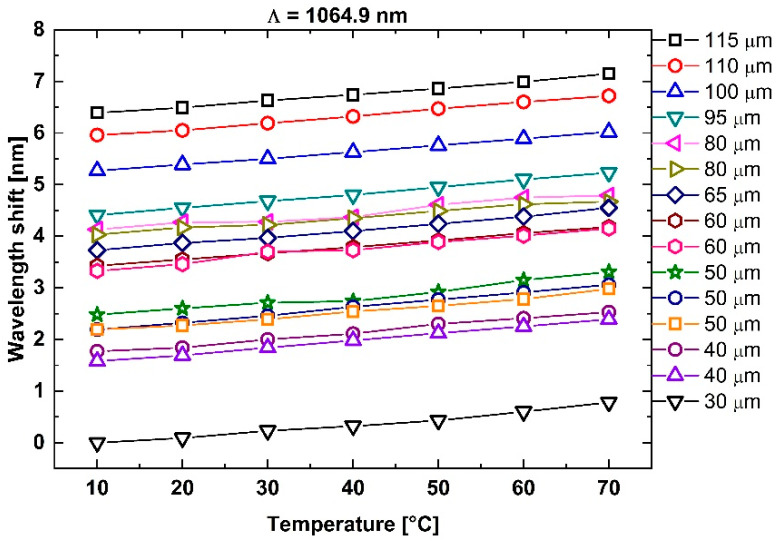
Bragg wavelengths shift as a function of temperature for tFBGs inscribed using the phase mask with pitch Λ_PM2_ = 1064.9 nm. Linear fits demonstrate a uniform thermal sensitivity of approximately 12.5 pm/°C across all taper diameters, confirming that the temperature response is dominated by silica’s thermo-optic and thermal-expansion coefficients.

Figure 7 shows the Bragg-wavelength shift (Δλ*_B_*) for tFBGs written with the pitch Λ*_PM_*_2_ = 1064.9 nm and different waist diameters, as a function of temperature in the 10–70 °C range. The symbols represent the experimental measurement points, while the lines are included only as visual guides. The extracted thermal-sensitivity coefficients and their corresponding average deviations for the tFBGs written with Λ_PM2_ = 1064.9 nm are summarized in Table 2. These results confirm that the thermal response remains nearly constant across all diameters, in agreement with the observations reported by Navarrete et al. [26], who also documented geometry-independent temperature sensitivity in tapered-fiber sensors dominated by the thermo-optic effect.

In the plotted results, the zero value on the *y*-axis simply corresponds to the smallest Bragg wavelength among the set of tFBGs and serves as a reference to display the relative wavelength shifts in the other gratings.

**Figure 8:**
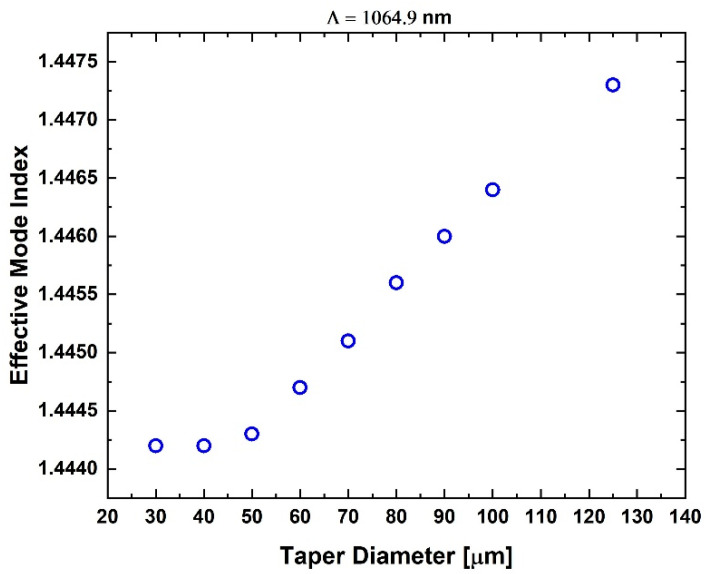
Effective refractive index *n*_eff_ as a function of tFBG waist diameter. The decreasing trend for thinner tapers reflects the weaker mode confinement in reduced cladding dimensions, consistent with guided-mode theory. This change directly influences the Bragg wavelength according to λ_B_ = 2Λ*n*_eff_.

At 10 °C, the FBG spectrum with the lowest Bragg wavelength was selected as the reference, and the relative wavelength shifts in the remaining tFBGs were evaluated with respect to this baseline. A reduction in the effective refractive index (*n*_eff_) of the Bragg gratings is observed as the fiber diameter decreases, in agreement with Equation (1) and as see in Figure 8. This trend results from weaker modal confinement in thinner waveguides, where the fundamental mode expands into the cladding and the surrounding medium. Such behavior confirms theoretical predictions for tapered fibers and micro/nanofiber waveguides and is consistent with previously reported experimental observations [10,11,34].

To avoid unintentional fiber stretching during the grating inscription process, the fiber was secured at both ends using magnetic holders, minimizing axial tension. This procedure helps maintain mechanical stability during UV exposure; however, complete elimination of residual eccentricity—particularly in very small diameters—is difficult to guarantee. Similar considerations regarding tension control, mechanical stability and sensitivity of tapered fibers to eccentricity effects have been discussed in previous works [12,15,30].

### 3.2. Strain Sensitivity

For applications involving strain measurements, it is necessary to ensure that the FBG used for temperature compensation remains strain-free, as the Bragg wavelength is simultaneously affected by both strain and temperature [1,2]. Alternatively, an additional FBG isolated from mechanical loading can be used to compensate for temperature variations, as commonly implemented in multi-parameter sensing schemes [5,13,35]. Conversely, for temperature measurements, the FBG must remain completely free of applied strain to guarantee accurate thermal characterization.

**Figure 9:**
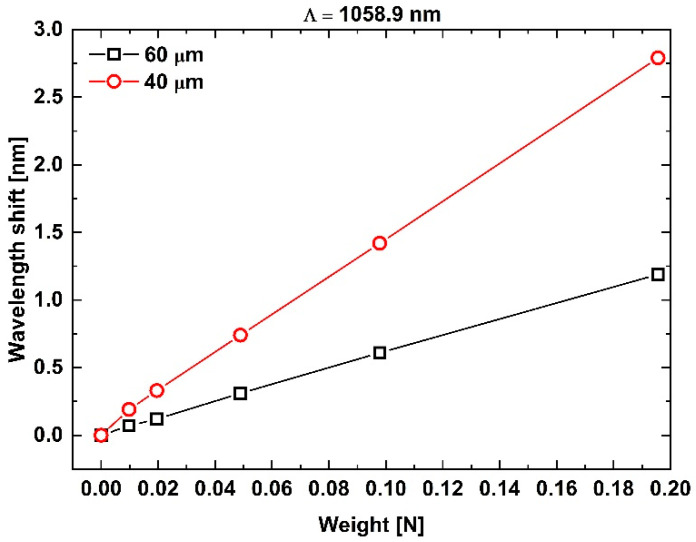
Bragg wavelengths shift Δλ_B_ as a function of suspended mass for two tFBGs inscribed using the phase mask with pitch 1058.9 nm. The nearly linear response and increasing slope for thinner diameters reveal the enhancement in strain sensitivity caused by reduced cross-sectional area.

Figure 9 shows the Bragg-wavelength shift (Δλ_B_) as a function of applied load for two tFBGs written with the pitch Λ_PM1_ = 1058.9 nm, each with a different waist diameter. As expected, the tFBG with the smaller diameter exhibits a larger wavelength shift under load, reflecting the inverse-square dependence of strain sensitivity on diameter, as described in previous studies on tapered fibers [13,30].

**Figure 10:**
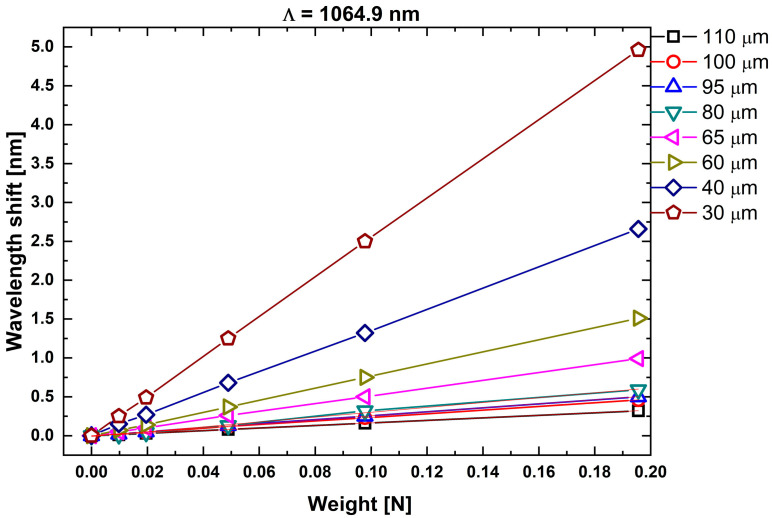
Bragg wavelengths shift Δ*λ_B_* under suspended mass for multiple tFBGs fabricated with pitch Λ_PM2_ = 1064.9 nm. The results confirm geometry-driven sensitivity enhancement and show consistent behavior across samples with different waist diameters.

Because the strain sensitivities of the tFBGs fabricated with the two different phase-mask pitches (1058.9 nm and 1064.9 nm) were found to be very similar for the 60 µm and 40 µm diameters—as shown in Figure 9 and Figure 10—no additional gratings were produced using the 1058.9 nm pitch. The strain sensitivities extracted from both phase-mask pitches are summarized in Table 3, confirming the expected scaling behavior and reinforcing that the grating pitch does not significantly modify the strain response in tapered fibers [13,30].

The tensile force applied over a homogeneous cylindrical body with varying cross-section produces higher strain in the thinner region, as expected from classical mechanics and consistent with the principles underlying FBG strain sensing [1,2]. Thus, the FBG located in this region becomes more sensitive to applied stress. This behavior also agrees with previous studies showing enhanced responsivity in diameter-reduced fibers such as etched FBGs and microfiber-based sensors [7,8,9,10,11,27].

Waveguide modeling was not performed since the focus of this work is experimental. Nevertheless, effective-index and sensitivity trends were estimated analytically from the Bragg condition and through taper geometry considerations, in agreement with established taper-shape and fiber-behavior models [16,30,31]. These analytical trends were validated by our experimental measurements.

This inverse-square dependence (S ∝ 1/D^2^) supports the assumption that localized stress magnifies the strain response in thinner waists. Similar principles have been reported in microfiber and tapered-fiber sensors, where reduced cross-sectional area significantly enhances sensitivity to external perturbations [10,11,27]

**Figure 11:**
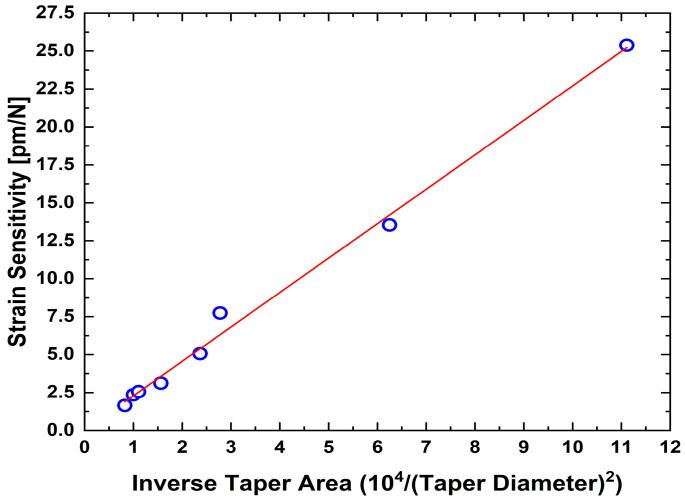
Strain sensitivity coefficient as a function of the inverse taper area 10^4^/D^2^. The strong linear correlation (R^2^ ≈ 0.9939) validates the empirical scaling law and demonstrates that strain sensitivity is primarily governed by the taper’s geometric reduction in cross-section.

The strain sensitivity of the tFBGs was found experimentally to scale inversely with the taper cross-sectional area, as expected from σ = F/A and ε ∝ 1/A. To demonstrate this dependence, Figure 11 plots the measured strain sensitivity (pm/N) versus 10^4^/D^2^ (where D is the taper diameter in μm). A linear fit yields:

S_load_ = (2.268 ± 0.030) (10^4^/D^2^) + (0.020 ± 0.12) (pm/N) (7)

with R^2^ = 0.9939. The excellent linear correlation confirms that the enhanced sensitivity arises primarily from the reduction in taper area (i.e., S∝1/A∝1/D^2^), consistent with previously reported sensitivity improvements in diameter-modified and tapered fiber structures [7,11,27,30].

## 4. Discussion

The thermal sensitivity coefficient of the tapered FBGs remained essentially invariant across all tested diameters, consistently falling within the 12.5–13.7 pm/°C range typically observed for conventional FBGs in untapered SMF-28 fibers [1,2]. Similar invariance in thermo-optic response has been reported in etched and diameter-modified FBGs, where the thermal coefficient is largely preserved despite strong geometric modifications [7,8]. In contrast, the strain sensitivity exhibited a strong dependence on geometry, increasing significantly as the waist diameter was reduced from 125 µm to 30 µm [10,11,27]. This decoupling between thermal and mechanical responses indicates that standard temperature-compensation strategies commonly applied to uniform FBGs can be readily extended to tapered configurations without requiring additional calibration procedures.

The enhancement in strain sensitivity with decreasing diameter follows the inverse-square dependence predicted by axial-stress theory [30,31] and is clearly reflected in Table 2. The highest sensitivity, 25.38 ± 0.06 pm/N, was achieved for the 30 µm taper, while the 110 µm taper exhibited a sensitivity of only 1.65 ± 0.01 pm/N. These results confirm that tapering provides an effective and predictable route for mechanical sensitivity enhancement while preserving the intrinsic thermal response governed by silica’s thermo-optic and thermal-expansion properties [1,2].

Additional insight into the influence of the inscription pitch was obtained by comparing tFBGs written using different phase masks but fabricated with identical waist diameters (40 µm and 60 µm). The nearly identical strain sensitivities measured in these samples demonstrate that the enhancement mechanism is dominated by geometric effects rather than inscription-related parameters, consistent with the phase-mask inscription principles discussed in [17]. The reduction in the effective refractive index as the cladding diameter decreases is consistent with the Bragg condition in Equation (1) and with previously reported taper-geometry behavior [12,13,15,16], which explains the blue shift observed in Figure 4. Despite these wavelength shifts, the thermal sensitivity remained constant across all diameters, reinforcing that geometry affects mechanical response but not the underlying thermo-optic behavior [1,7,8].

The fabrication method adopted in this work—UV phase-mask inscription on telecom-grade SMF-28, combined with a controlled resistive-heating tapering process using a Ni-Cr micro-heater—provides a highly scalable and reproducible approach. The phase-mask technique is well established for high-uniformity and repeatable FBG writing [17], while the use of a resistive micro-heater enables precise thermal conditioning of the fiber and accurate control of taper geometry, as demonstrated in previous taper-fabrication studies [12,15,28,29]. Unlike flame-based techniques, the resistive heater ensures a stable and uniform thermal zone, enabling precise control of the waist diameter and smooth adiabatic transitions [16]. This method also avoids the complexity and cost associated with femtosecond-laser inscription or chemical etching while maintaining full compatibility with standard OSA-based interrogation systems. Therefore, the proposed fabrication route is particularly attractive for large-scale deployment in industrial sensing and structural-health-monitoring environments [18,33].

A detailed comparison with representative results from the literature is presented in Table 4. Although direct quantitative comparisons are complicated by differences in fabrication techniques, cladding geometries, and reporting metrics (pm/N vs. pm/µε), two general trends consistently emerge: (i) strain sensitivity increases sharply as the effective cross-sectional area is reduced, and (ii) thermal sensitivity remains primarily governed by the material properties of silica unless the fiber enters the deep-microfiber regime [1,2,7,10,11,27]. For example, chemically etched and regenerated FBGs [7,8], half-etched fibers [9], and microfiber Bragg devices [10,11] all exhibit enhanced strain responses, but their thermal behavior can vary considerably depending on the degree of modal confinement and environmental coupling.

Our measurements align with these trends: the maximum strain sensitivity of 28.84 pm/µε (equivalent to 25.38 pm/N) for the 30 µm taper is substantially higher than the ≈1.2 pm/µε typical of untapered SMF-28 FBGs [1,2], yet the thermal sensitivity remains close to 12.5 pm/°C, consistent with the characteristic thermo-optic response of silica and similar to values reported for etched and regenerated FBGs [7,8]. This indicates that the mode remains sufficiently confined within the silica structure despite the reduced cross-section.

This contrasts with sub-10 µm microfiber Bragg gratings, where significant modal leakage can cause strong dependence on the external medium and altered thermal responses [10,11,27,36]. In this deep-microfiber regime, strong evanescent-field interaction and reduced structural rigidity led to both enhanced sensitivity and higher environmental susceptibility [27,30,31,36]. As such, while extrapolation of the strain–diameter relation below ~10 µm is theoretically straightforward from axial-mechanics considerations [30,31], experimental verification would require dedicated modeling and careful mechanical handling strategies due to the fragility and environmental sensitivity of microfibers.

The combination of predictable strain-scaling behavior and robust thermal invariance opens promising opportunities for next-generation tapered-FBG sensing systems. Future work will benefit from finite-element modeling of stress and optical-mode distributions to refine analytical predictions [12,16,27], as well as from high-precision calibration using micro-tensile instrumentation for thin fibers [30]. The distinct Bragg wavelengths associated with different taper diameters also offer a natural basis for wavelength-division multiplexing, enabling compact sensor networks for distributed or quasi-distributed monitoring in composite structures and civil infrastructure [18,33].

Overall, this study demonstrates that tapering standard SMF-28 fibers provides a low-cost, highly reproducible, and telecom-compatible approach to significantly enhance strain sensitivity without sacrificing thermal stability. These findings establish a strong experimental and theoretical foundation for the development of compact, high-performance sensing platforms based on tapered FBG technology [12,15,27,36].

## Figures and Tables

**Table 1:** The diameters and numbers of the tapers.

tFBG Cladding Diameter [µm]	Numbers Produced
115	1
110	1
100	1
95	1
80	2
65	1
60	2
50	3
40	2
30	1

**Table 2:** Thermal Sensitivity for tFBGs with Λ_PM_ = 1064.9 nm.

Taper Diameter (µm)	Thermal Sensitivity (pm/°C)
115	12.54 ± 0.40
95	13.68 ± 0.77
60	13.57 ± 0.40
50	13.04 ± 0.09
30	12.71 ± 0.24

**Table 3:** The strain sensitivity coefficients.

**Λ_PM1_ = 1058.9 nm**			
**Diameter [µm]**	**S_load_ [pm/N] ± σ**	**S_ε_ [pm/µε] ± σ**	**S_temp_ [pm/°C] ± σ**
60	6.08 ± 0.05	2.99 ± 0.02	
40	14.10 ± 0.14	15.60 ± 0.15	
**Λ_PM2_ = 1064.9 nm**			
110	1.65 ± 0.015	2.41 ± 0.02	13.07 ± 9.27
100	2.36 ± 0.020	3.35 ± 0.03	12.54 ± 3.17
95	2.56 ± 0.021	3.62 ± 0.03	13.68 ± 5.64
80	3.12 ± 0.100	4.55 ± 0.15	11.68 ± 9.80
65	5.06 ± 0.030	6.25 ± 0.04	13.39 ± 3.40
60	7.74 ± 0.030	11.33 ± 0.04	13.57 ± 2.93
40	13.54 ± 0.060	14.99 ± 0.07	13.29 ± 2.62
30	25.38 ± 0.060	28.84 ± 0.05	12.71 ± 1.85

**Table 4:** Comparison of strain and temperature sensitivity of tapered, etched and microfiber FBGs reported in the literature.

Reference	Method/Structure	Waist (µm)	Strain Sensitivity	Temperature Sensitivity	Notes
This work (2025)	UV phase-mask tFBG on SMF-28	30	28.84 pm/µε (or 25.38 pm/N)	12.5 pm/°C	Stable, low-cost, reproducible
Wang et al. (2014) [8]	Etched + regenerated FBG	~12	~6.8 pm/µε (enhanced vs. standard FBG)	~10.3 pm/°C	Chemical etching + regeneration
Zhang et al. (2018) [14]	Femtosecond written tapered FBG	25	Not strain-focused	9.6–12.3 pm/°C	Temperature sensor; fs-laser inscription
Kou et al. (2012) [10]	Microfiber Bragg	2–10	Up to ~150–500 pm/µε depending on device	15–25 pm/°C (varies)	Microfiber regime: strong evanescent field
Dey et al. (2021) [9]	Half-etched FBG	~12	1.96 nm/N = 1960 pm/N	~10–12 pm/°C	Very high force sensitivity using cross-section reduction

## Data Availability

The original contributions presented in this study are included in the article. Further inquiries can be directed to the corresponding author.

## Abbreviations

The following abbreviations are used in this manuscript:

ASE	Amplified Spontaneous Emission
FBG	Fiber Bragg Grating
FEM	Finite Element Modeling
LPG	Long-Period Grating
OSA	Optical Spectrum Analyzer
PID	Proportional-Integral-Derivative
PM	Phase Mask
SHM	Structural Health Monitoring
SMF	Single-Mode Fiber
SMF-28	Standard Telecom Single-Mode Fiber (G.652)
tFBG	Tapered Fiber Bragg Grating
TEC	Thermoelectrical Cooler
UV	Ultraviolet
ΛPM	Phase Mask Pitch
